# Two-Dimensional, M-Mode, and Doppler Echocardiographic Reference Intervals in Adult Chickens (*Gallus gallus domesticus*) of Hobby Flock Breeds

**DOI:** 10.3390/ani16091308

**Published:** 2026-04-24

**Authors:** Hillary K. Hammond, Brian G. Barnett, Nicole Sallaberry-Pincheira, Laura Burns, Eva Patnoude, Amanda E. Coleman

**Affiliations:** 1 Department of Small Animal Medicine and Surgery, College of Veterinary Medicine, University of Georgia, Athens, GA 30602, USA; bgray90@uga.edu (B.G.B.); nicole.sallaberry@uga.edu (N.S.-P.); laurab@uga.edu (L.B.); eva.patnoude@uga.edu (E.P.); mericksn@uga.edu (A.E.C.); 2 Unidad de Rehabilitacion de Fauna Silvestre, Escuela de Medicina Veterinaria, Facultad de Ciencias de la Vida, Universidad Andres Bello, Republica 440, Santiago 8370146, Chile

**Keywords:** echocardiogram, chicken, reference interval, allometric scaling, cardiovascular

## Abstract

The keeping of companion chicken flocks has recently grown in popularity, increasing the demand for advanced veterinary care. The clinical utility of echocardiography is currently limited by a lack of established reference intervals for adult chickens of non-commercial breeds. Therefore, the objective of this prospective observational study was to establish echocardiographic reference intervals for adult chickens of non-commercial breeds. Healthy adult chickens (*n* = 126) of both sexes and various breeds were administered intramuscular butorphanol (2 mg/kg) and midazolam (2 mg/kg) before undergoing echocardiography by a board-certified veterinary cardiologist. Using nonparametric methods, 95% reference intervals were determined, with the 2.5th (90% CI) and 97.5th (90% CI) percentiles serving as the lower and upper limits, respectively. The effect of body weight on linear cardiac dimensions was evaluated by regression analysis, and allometric equations scaled to body weight were derived for each variable. Intraclass correlation coefficients were used to quantify echocardiographic intra- and interoperator repeatability and intra- and interobserver measurement agreement. Body weight was a significant but not strongly correlative co-variate, and 95% prediction intervals for linear dimensions were determined by allometric scaling. Echocardiographic reference intervals were established from 126 chickens of non-commercial breeds.

## Simple Summary

The goal of this study was to determine normal heart ultrasound (echocardiography) measurements in adult chickens not raised for commercial production. A total of 126 healthy adult chickens of different breeds were sedated to facilitate an echocardiogram performed by a veterinary cardiologist. The data were used to establish reference intervals and to determine which measurements were affected by body weight. The study also evaluated how reliable these measurements are when obtained and measured by different people on different days. Overall, this study establishes normal echocardiographic values for adult chickens of non-commercial breeds. These results are valuable because as more people keep chickens as pets, veterinarians need reliable reference information to properly diagnose and manage heart disease in these birds.

## 1. Introduction/Objectives

Diagnostic advancements from the field of human cardiology have been adapted for use in a variety of veterinary species. The first descriptions of echocardiography (cardiac ultrasound) were by Edler and Hertz in the 1950s [1]. Subsequently, veterinary studies using echocardiography were first published in the late 1970s [2] and the development of standardized echocardiographic methods in dogs [2,3,4] and cats [5] preceded the development of echocardiographic reference intervals in these and other mammalian species [6,7,8,9,10,11,12,13]. Interest in the use of this technology for non-human species has been driven by the advantages it offers as a safe, non-invasive tool that facilitates accurate and comprehensive evaluation of the heart’s structure and function, as well as measurement or estimation of numerous hemodynamic parameters. As such, echocardiography currently represents an invaluable tool for the assessment of cardiovascular health and pathology and is commonly used in clinical veterinary practice.

Echocardiography has also been used in avian species, for which standardized echocardiographic techniques [14,15,16,17,18,19,20] and reference intervals have been described [16,19,21,22,23]. Antemortem diagnoses of round heart disease [24]; pulmonary hypertension syndrome [25,26]; pericardial, myocardial, and congenital heart diseases [14,15,18,27]; and infective endocarditis [14,15,18], among other pathological processes, have been possible through the application of this technology in birds.

In recent years, the keeping of companion chicken (*Gallus gallus domesticus*) flocks has significantly grown in popularity [28]. In 2025, approximately 11 million American households reported having backyard chickens, a 28% increase compared to 2023 [29]. In addition, chickens are considered the third-most popular pet in many countries [28]. Two-thirds of backyard chicken owners in the United States view their chickens as pets [29]. This, as well as recent case reports highlighting the application of advanced diagnostics and treatments for pet chickens—including coelioscopy [30], transvenous occlusion of patent ductus arteriosus [27], and ingluviotomy [31]—suggests that demand for individualized veterinary medical care for these animals is increasing.

Reliable reference intervals facilitate the accurate interpretation of diagnostic tests. While such intervals have been suggested for echocardiographic parameters in chickens [23,25,32], most have been derived from small samples of production poultry that are generally young (i.e., 5–7 weeks of age). The fast growth of production chicken lines distinguishes them from typical backyard flock breeds and can result in differences in cardiac development between groups and body-mass-to-heart-mass mismatch [14], limiting the usefulness of existing reference intervals. Additionally, data from at least 120 individuals are recommended for determination of population-based reference intervals in veterinary medicine [33]; however, to date, the largest sample used to generate adult echocardiographic reference intervals comprised 29 birds. Finally, relevant studies have focused almost exclusively on left ventricular dimensions, without reporting parameters related to left atrial size, ventricular systolic and diastolic performance, or blood velocity.

While there is a need for comprehensive and widely applicable reference intervals that are specific to adult chickens of backyard breeds and that have been generated in a manner consistent with published guidelines, doing so involves the challenge of accounting for variability in body size among these breeds. One approach to this challenge, which is more practical than generating breed-specific reference intervals, involves normalizing measurements to body weight [34,35,36]. The objective of this study was to establish body weight-independent reference intervals and assess repeatability for several echocardiographic measurements in sedate healthy chickens of common backyard flock breeds.

## 2. Materials and Methods

Animals:

One hundred and forty-six chickens from an “exotic breed” flock kept at the University of Georgia Poultry Research Center were screened for use in this study. A minimum sample size of 120 subjects was based on the Clinical and Laboratory Standards Institute guidelines for establishing reference intervals by nonparametric methods with 90% confidence intervals around the limits. All screened chickens were between 1 and 2 years of age and weighed between 0.5 and 4.0 kg. Chickens were group housed in a large temperature-controlled room measuring 40 by 60 feet, with individuals of each breed placed in the same pen, measuring eight by ten feet, with pine shavings covering the floor. Chickens were fed a stock diet made by the University of Georgia’s feed mill.

Included chickens were deemed healthy based on the findings of physical, electrocardiographic, and echocardiographic examinations. Chickens with heart murmurs were not excluded unless they had clear echocardiographic evidence of heart disease. Data from chickens with one or more of the following were excluded from normal reference interval determination: (1) body condition score [37] less than 2 of 5, (2) evidence of systemic illness based on physical examination, (3) electrocardiographic evidence of supraventricular or ventricular ectopy, third degree atrioventricular block, or second degree atrioventricular block persisting despite reversal of sedation, (4) obvious cardiac valve thickening, (5) valvular insufficiency severity greater than mild, (6) congenital cardiac disease, or (7) pericardial effusion.

Echocardiographic image acquisition:

All examinations were performed in June and July 2024. On the morning of evaluation, chickens were transported in groups of 10 to 15 from their housing facility at the University of Georgia Poultry Research Center to a temperature-controlled room at the Veterinary Teaching Hospital, where they were placed in a wire crate with access to fresh water. Chickens were allowed to acclimate for at least five minutes and up to five hours prior to handling. Each chicken underwent body weight measurement and physical examination performed by a veterinary resident in zoological medicine or in cardiology, or by a board-certified veterinary cardiologist. Individual chickens were then moved into a darkened isolation crate for at least five minutes immediately after being administered a combination of butorphanol (2 mg/kg) and midazolam (2 mg/kg) into a pectoral muscle. Prior to and at five-minute intervals after drug administration, heart rate, respiratory rate, compliance, and sedation depth were monitored and recorded; this was continued throughout the recovery period until animals were able to ambulate [38,39].

**Figure 1:**
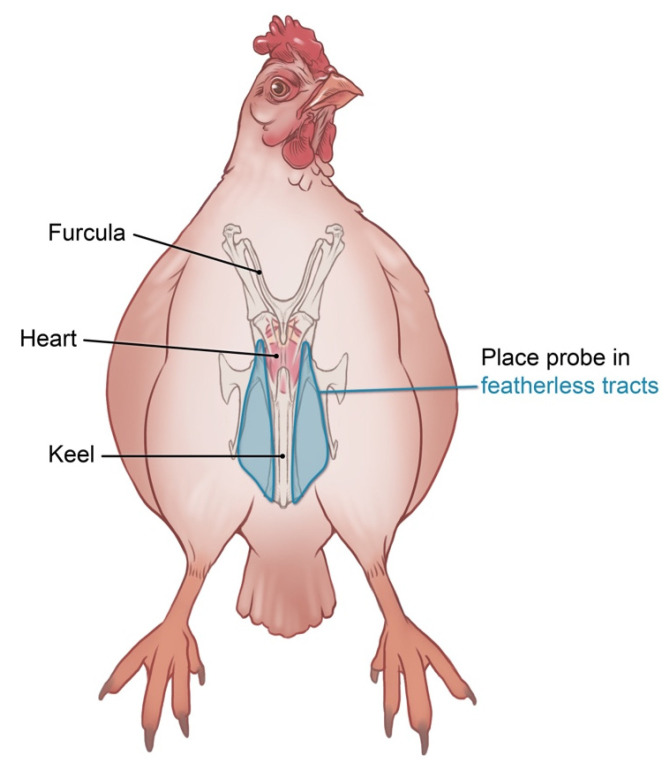
Schematic representation of the featherless tracts through which acoustic windows can be accessed for echocardiography in the chicken.

Full echocardiographic studies, including two-dimensional (2D), M-mode (MM), and Doppler echocardiography, were performed by a board-certified cardiologist (H.K.H.) using an ultrasound system (Philips Healthcare iE33, Philips North America Corporation, Andover, MA, USA) equipped with S8-3 and S12-4 phased array sector transducers. Still images and cine loops were recorded from right and left parasternal imaging planes. Chickens were gently restrained in an upright standing or “seated” position. The probe was positioned one to three cm lateral to the keel at the approximate level of the stifle within a featherless tract (Figure 1). Image quality was optimized using a combination of 70% isopropyl alcohol and ultrasonic gel as previously described [16].

Acquisition of images was standardized, using a protocol that was modified from standards used in dogs and cats [40]. Still images and cine loops from 5 s were stored for later analysis using an off-cart workstation and measurement software (Syngo Dynamic Workplace Version VA40, Siemens Medical Solutions, Malvern, PA, USA). Synchronized electrocardiography was not utilized during the echocardiogram to minimize stimulation and stress, as has been reported in other avian studies [19,20].

**Figure 2:**
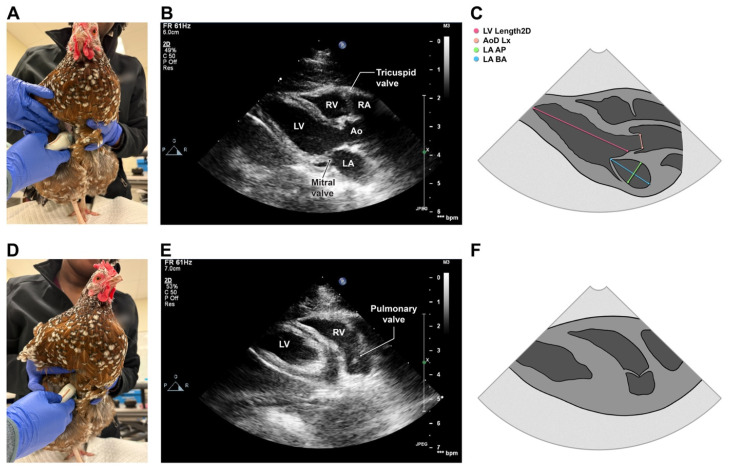
Probe placement (**A**,**D**), representative echocardiographic images (**B**,**E**) and landmarks for select echocardiographic measurements (**C**,**F**) taken from the right parasternal long-axis (**A**–**C**) and right parasternal oblique long-axis (**D**–**F**) views in 126 healthy chickens. LV, left ventricle; RV, right ventricle; RA, right atrium; LA, left atrium; Ao, aorta; LV length 2D, left ventricular length measured from the aortomitral curtain to the blood–endocardium interface of the left ventricular apex; LA-AP, antero-posterior dimension of the left atrium; LA-BA, basiloapical dimension of the left atrium; AoD Lx, aortic diameter. Photos (**A**,**D**) taken by authors.

The ultrasound probe was first positioned on the right side of the keel with its reference marker directed cranially to obtain a right parasternal five-chamber long-axis view that optimized the length of the left ventricular chamber and allowed simultaneous visualization of the atria, ventricles and aortic root (Figure 2A,B). Color Doppler imaging was performed over the left atrioventricular (mitral), aortic, and right atrioventricular valves to assess for insufficiency. Severity of insufficiency was classified as trace, mild, moderate, or severe using the jet size, with mild insufficiency occupying less than 20% of the atrium, moderate 20 to 40%, and severe as greater than 50% [41]. The probe was then tilted cranially and rotated clockwise slightly to obtain a right parasternal “oblique long-axis” view that optimized the right ventricular outflow tract and pulmonary valve (Figure 2D,E). Color Doppler imaging was performed over the pulmonary valve to assess for insufficiency. Continuous-wave (CW) and pulsed-wave (PW) spectral Doppler recordings were made by optimizing cursor line-up with right ventricular outflow; for PW, a sampling volume was placed at the level of the pulmonary valve.

**Figure 3:**
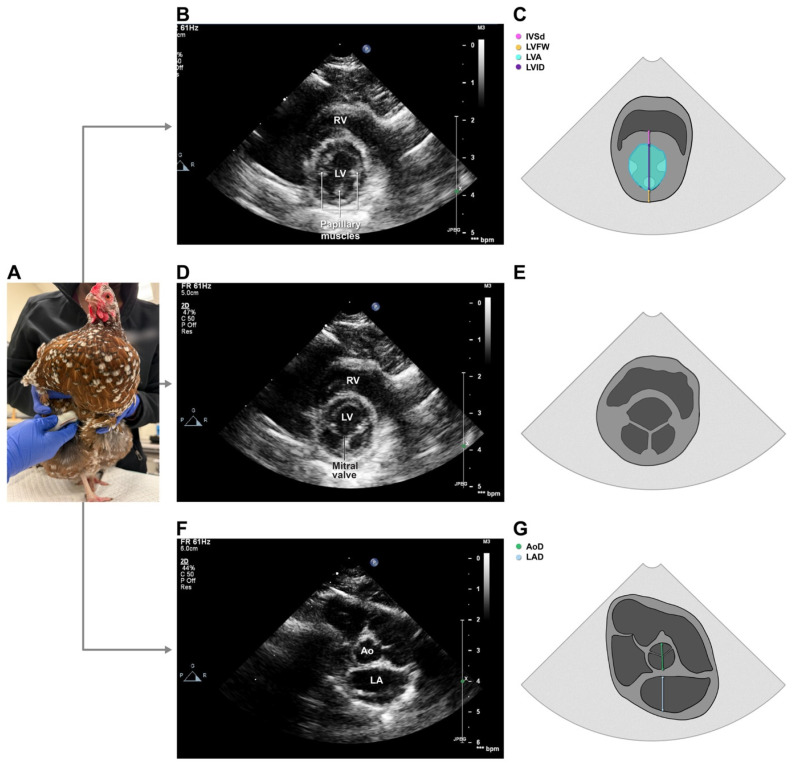
Probe placement (**A**), representative echocardiographic images (**B**,**D**,**F**) and landmarks for select echocardiographic measurements (**C**,**G**) taken from right parasternal short-axis views at the level of the left ventricular papillary muscles (**B**,**C**), mitral valve (**D**,**E**) and aortic valve (**F**,**G**) in 126 healthy chickens. LV, left ventricle; LA, left atrium; Ao, aorta; RV, right ventricle. Photo (**A**) taken by authors.

An approximately 90 degrees clockwise (i.e., toward the keel) rotation of the probe allowed acquisition of right parasternal short-axis (i.e., perpendicular to the long axis of the heart) images, which were generated by “fanning” the probe from the apex to the base of the heart (Figure 3). At the level of the left ventricular papillary muscles, using a view that optimized the roundness of the left ventricular cavity and maintained simultaneous visualization of the right ventricular cavity, short-axis 2D cine loops and 2D-guided M-mode images were stored. This was repeated at the level of the mitral valve leaflets. M-mode images were generated by placing the cursor perpendicular to the interventricular septum and left ventricular free wall and bisecting the left ventricular cavity. Color Doppler imaging was also performed over the mitral valve to assess for insufficiency. At the level of the aortic valve, using a view that allowed visualization of the commissures of the aortic valve cusps during diastole, 2D cine loops and 2D-guided M-mode images—the latter taken with the cursor bisecting the aortic annulus—were obtained. Color Doppler imaging was performed over the aortic, right atrioventricular and pulmonary valves to assess for insufficiency. Finally, PW and spectral Doppler recordings of right ventricular outflow were generated in the same manner as described above for the oblique long-axis view.

**Figure 4:**
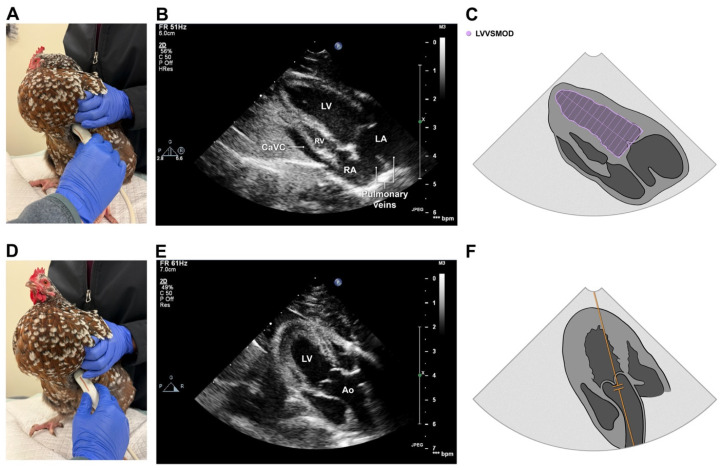
Probe placement (**A**,**D**), representative echocardiographic images (**B**,**E**) and landmarks for select echocardiographic measurements (**C**,**F**) taken from the left parasternal long-axis (**A**) and left parasternal outflow long-axis (**E**) views in 126 healthy chickens. LV, left ventricle; RV, right ventricle; RA, right atrium; LA, left atrium; Ao, aorta; CaVC, caudal vena cava; LVVSMOD, tracing the endocardium-blood interface from the mitral valve hinge points to the endocardial border of the LV apex. Photos (**A**,**D**) taken by authors.

The ultrasound probe was then positioned on the left of the keel with its reference marker directed cranially to obtain a left parasternal four-chamber view that optimized the length of the left ventricular chamber and allowed simultaneous visualization of both atria and both ventricles (Figure 4A,B). From this plane, color Doppler imaging was performed over the mitral and right atrioventricular valves to assess for insufficiency and to guide PW spectral Doppler recordings of left ventricular inflow. The latter were made by placing a sampling volume within the left ventricle at the level of the open mitral valve leaflet tips. The probe was then rotated clockwise and angled toward the dorsal midline to obtain a left ventricular outflow view that optimized the outflow tract and aortic valve. Color Doppler imaging was performed over the aortic valve to assess for insufficiency. Continuous-wave and PW spectral Doppler recordings were made by optimizing cursor line-up with left ventricular outflow and, for PW, placing a sampling volume at the level of the aortic valve (Figure 4D).

Following echocardiography, an electrocardiogram was performed with each chicken gently restrained in a “seated” position, with the wings slightly retracted to facilitate electrode placement [42]. Then, reversal of sedation was achieved by administering intramuscular flumazenil at a dose of up to 0.13 mg/kg, tailored to each bird’s level of sedation. Chickens were monitored during recovery until they regained the ability to ambulate. Once ambulatory, they were placed in a warmed crate with access to fresh water and allowed to recover further. The birds were transported to their housing facility at the end of the day.

Echocardiographic measurements and calculations:

All measurements were performed off-line by a single investigator (B.G.B.). For each parameter, 3–5 values from separate cardiac cycles were recorded, the average of which was used for statistical analyses. For linear 2D cardiac chamber measurements, an inner-edge-to-inner-edge (i.e., at the blood–endocardium interface) measurement technique was used, and for linear M-mode cardiac chamber measurements, a leading-edge-to-leading-edge measurement technique was used. For area and volume cardiac chamber measurements, the blood–endocardium interface was traced, and the left ventricular papillary muscles were ignored.

The 2D right parasternal long-axis 5-chamber view was used to for linear measurements of the left ventricle (LV), left atrium (LA) and aorta (Ao). Left ventricular length (LVL) was measured from the aorto-mitral curtain to left ventricular apex at end-diastole (LVLd^2DE^) and end-systole (LVLs^2DE^); these measurements were used to calculate LVL fractional shortening (LVL-FS%) as (LVLd^2DE^ − LVLs^2DE^)/(LVLd^2DE^) × 100. End-systolic LA dimensions, measured in anteroposterior (LA-AP) and basiloapical (LA-BA) orientations, were measured on the frame immediately prior to mitral valve opening, with LA-BA extending from the coaptation point of the mitral valve leaflets to the roof of the left atrium and bisecting the chamber, and LA-AP extending from the interatrial septum to the left atrial free wall parallel to the mitral valve annulus and bisecting the chamber (Figure 2C). The aortic diameter in long axis (AoD^2DE,Lx^) was measured during early systole when valve excursion was at its greatest, from leaflet tip to leaflet tip, or from valve hinge point to hinge point when leaflet tips could not be adequately visualized (Figure 2C).

The 2D right parasternal oblique long-axis view was used to measure pulmonary valve diameter (PVD^Lx^) in early systole in a manner similar to AoD^2DE,Lx^. Pulsed-wave spectral Doppler recordings from this imaging plane were used to measure peak transpulmonary valve velocity (PV Vmax^Lx^), as well as transpulmonary velocity-time integral (PV VTI^Lx^), acceleration time (PV-AT^Lx^) and ejection time (PV-ET^Lx^), using previously described methods [43]. The ratio of PV-AT^Lx^ and PV-ET^Lx^ (PV AT:ET^Lx^) was also calculated.

The 2D right parasternal short-axis view taken at the level of the LV papillary muscles was used to obtain end-diastolic and end-systolic linear and area measurements of the LV. For these, end-diastole and end-systole were defined as the time of maximum and minimum left ventricular chamber dimensions, respectively. Interventricular septal wall (IVS) thickness, left ventricular internal dimension (LVID), and left ventricular free wall (FW) thickness at end-diastole (IVSd, LVIDd and LVFWd, respectively) and end-systole (IVSs, LVIDs and LVFWs, respectively) were obtained from both 2D and M-mode images. Left ventricular fractional shortening was calculated for each of 2D- (FS%^2DE^) and M-mode-derived (FS%^MM^) measurements, where FS% = (LVIDd − LVIDs)/LVIDd × 100. Left ventricular area was measured on 2D images at end-diastole (LVAd) and end-systole (LVAs) (Figure 3B) and used to calculate LV fractional area change (LV FAC%) as (LVAd − LVAs)/LVAd. M-mode images taken from the right parasternal short-axis view at the level of the mitral valve leaflets were used to measure E-point-to-septal separation (EPSS), defined as the distance between the IVS and the mitral valve at its point of greatest excursion during early diastolic filling, as previously described [41].

The right parasternal short-axis view taken at the level of the aortic valve was used for 2D and M-mode linear measurements of the aortic root (AoD^2DE,Sx^, AoD^MM^) and left atrium (LA^2DE,Sx^, LA^MM^), from which left-atrial-to-aortic root ratios (LA: Ao^2DE^ and LA:Ao^MM^) were calculated. Two techniques, one proposed by Rishniw and Erb [44] and the other described by Hansson et al. [45], were used for 2D linear measurements (Figure 3G). This view was also used to measure the diameter of the pulmonary valve annulus (PVD^Sx^) in early systole in a manner similar to AoD^2DE,Lx^. Pulsed-wave spectral Doppler recordings from this imaging plane were used to measure peak transpulmonary valve velocity (PV Vmax^Sx^), as well as transpulmonary velocity-time integral (PV VTI^Sx^), acceleration time (PV-AT^Sx^) and ejection time (PV-ET^Sx^), using previously described methods [43]. The ratio of PV-AT^Sx^ and PV-ET^Sx^ (PV AT:ET^Sx^) was also calculated.

The 2D left parasternal long-axis 4-chamber view was used for volumetric measurements of the LV chamber at end-diastole (LVVd^SMOD^) and end-systole (LVVs^SMOD^) using Simpson’s method of disks (SMOD), as previously described [46]. For these, the LV area was measured by tracing the endocardium-blood interface, maximum length was taken from the midpoint of a line connecting the anterior and posterior mitral valve hinge points to the endocardial border of the LV apex (Figure 4C), and LV volume was calculated automatically by the machine software. Left ventricular ejection fraction (EF^SMOD^%) was calculated as (LVVd^SMOD^ − LVVs^SMOD^)/LVVd^SMOD^. Spectral Doppler recordings from this view were also used to measure peak mitral valve inflow velocity (MV Vmax).

Finally, PW spectral Doppler recordings from the left parasternal LV outflow view were used to measure peak transaortic valve velocity (AV Vmax), velocity-time integral (AV-VTI), acceleration time (AV-AT) and ejection time (AV-ET), from which the ratio of AV-AT and AV-ET (AV AT:ET) was also calculated. Continuous wave spectral Doppler recordings from the same view were used to measure peak left ventricular outflow tract velocity (LVOT Vmax) and velocity-time integral (VTI).

Evaluation of measurement agreement and repeatability:

Intra-operator, day-to-day repeatability was performed by randomly selecting 10 chickens to undergo a second echocardiographic examination by the original sonographer (H.K.H), at least 48 h after the initial assessment. Two additional sonographers also performed echocardiographic assessments of those chickens to evaluate inter-operator variability. To assess intra-observer measurement agreement (variability), the same masked investigator (B.G.B.) performed all echocardiographic measurements on 10 randomly selected studies on three different occasions, each separated by at least one week [36]. To assess inter-observer variability, three masked investigators (B.G.B, H.K.H., A.E.C.) performed all measurements on the same 10 randomly selected studies.

Statistical analysis:

Statistical analyses were performed using commercially available computer software (MedCalc Statistical Software Version 23.4.0). All echocardiographic measurements and calculations were tabulated, visually inspected using dot plots, and tested for normality of distribution using a Shapiro–Wilk test. Data were tested for statistical outliers using Tukey’s method. Statistical outliers were examined and only considered for removal if measurement or clerical error was thought to have occurred.

The 95% reference intervals were determined according to the recommendations of the Clinical and Laboratory Standards Institute. The 2.5% and 97.5% points of distribution were calculated using nonparametric percentile method when the reference sample population exceeded 120 subjects and by the robust method when the reference sample population was between 100 and 120 subjects via bootstrapping [33].

Linear regression analysis was performed after logarithmic transformation of the data to evaluate the relationship of the echocardiographic measurements and body weight using allometric scaling. The equation Y = ax^b^ was used to determine the proportionality constant (a) and scaling exponent (b) for each measurement as previously described [34,35,36]. The antilogarithm of the intercept of the resultant line is the constant *a*, and the slope of the resultant line gives the constant *b*. *X* represents the body weight of the chicken, and Y represents the value of the echocardiographic measurement. Prediction intervals were determined for measurements that demonstrated significant (*p* < 0.05) and at least weak correlation (r > 0.2) with body weight. Due to the large sample size, prediction intervals were calculated using *a_c_* = 10 ^(log(*a*) ± *t х Sx,y*)^, where *a_c_* is the calculated proportionality constant, *a* is the proportionality constant from the linear regression equation, *t* is the desired Student’s t statistic for n − 2 degrees of freedom and the desired degree of confidence, and *S_x,y_* is the standard error of the Y estimate from linear regression.

Intraclass correlation coefficient (ICC) was used to quantify echocardiographic intraobserver, interobserver, intraoperator, and interoperator measurement agreement. For ICC calculations, a 2-way single measures mixed effect model (where all the subjects are measured by the same observers) for absolute agreement was selected [47]. Agreement was considered poor for values of 0–0.2, fair for values of 0.21–0.40, moderate for values of 0.41–0.6, substantial for values of 0.61–0.8, and almost perfect for values of 0.81–1 [48,49]. Significance level was set at *p* < 0.05.

## 3. Results

One hundred and forty-six chickens underwent physical, echocardiographic, and electrocardiographic examinations. Data from twenty-one examinations (14.3%) were excluded for the following reasons: greater than mild valvular insufficiencies on echocardiogram (*n* = 7), supraventricular arrhythmia (*n* = 4), inadequate body condition score (*n* = 4), structural heart disease (*n* = 4), ventricular ectopy (*n*= 1), and unintentional repeat evaluation of the same individual (*n* = 1). Detected heart diseases included one case each of infective endocarditis, ventricular septal defect, non-specific mitral valve abnormality, and fixed left ventricular outflow tract obstruction.

The final reference population consisted of 126 chickens, 89 (70.1%) of which were female. Median (min, max; IQR) body weight of included chickens was 1.88 kg (0.52 kg, 3.78 kg; 1.56–2.32 kg). Males were significantly heavier than the females (*p* < 0.0001). Fourteen breeds were represented: Leghorn (*n* = 44), Athens Random Breed (*n* = 42), Cochin Bantam *(n* = 8), Cochin (*n* = 7), mixed *(n* = 7), Ameraucana (*n* = 3), Naked Neck (*n* = 3), Barred Rock (*n* = 3), Silver Spangled Hamburg (*n* = 2), Wyandotte (*n* = 2), Rhode Island Red (*n* = 2), Polish Top Hat (*n* = 2), and White Silkie (*n* = 1).

Echocardiographic images were successfully obtained in all included chickens with no observed adverse effects. No obvious measurement errors were retrospectively identified for statistical outliers; therefore, no measurements were excluded. Mean ± SD time between the administration of sedative agents and start of echocardiography was 12.1 ± 5.4 min. Echocardiographic study duration, taken as the time between acquisition of the first and last images, was 7.3 ± 0.2 min. The average heart rate as determined by a six-lead electrocardiogram obtained within 2 min of completion of echocardiogram was 274 ± 64 beats per minute [42].

Mitral valve insufficiency, most commonly identified from the right parasternal long-axis view, was noted in 30 (23.8%) of 126 chickens, for which severity was trace (*n* = 17), trace-to-mild (*n* = 1), or mild (*n* = 13). Aortic valve insufficiency, identified with equal frequency in the right parasternal short- and long-axis views, was present in 14 (11.1%) of 126 chickens, for which severity was trace (*n* = 9) or mild (*n* = 5). Right atrioventricular valve insufficiency, most frequently appreciated in the left parasternal four-chamber view, was present in 6 (4.8%) of 126 chickens (trace in *n* = 4; mild in *n* = 2). Adequate 2D imaging of the pulmonary valve from any view was possible in only 32 (25.3%) of 126 chickens; of these 32, two had trace pulmonary valve insufficiency.

Descriptive statistics and proposed reference intervals for echocardiographic measurements are presented in Table 1. Spectral Doppler from the right ventricular outflow is provided in Table A1 due to lack of confirmed line up with pulmonary valve annulus. A summary of the linear regression data describing the relationship between log10 of selected linear chamber measurements and log10 body weight are presented in Table 2. All linear cardiac measurements presented demonstrate a significant (all *p* < 0.005) and at least weak (r > 0.2) correlation with body weight. All scaling exponents for linear measurements were close to the theoretical value of 1/3. Mean values and 95% prediction intervals are provided for 0.5 kg intervals over a range of 0.5–5 kg in Table 3.

Intraobserver and interobserver measurement agreement and intraoperator and interoperator repeatability data are presented in Table 4. Intraobserver measurement agreement was at least substantial for all measurements except IVSd^MM^, LVFWd^MM^, AoD^MM^, and LAD^MM^. Interobserver agreement was at least substantial for all measurements except LVFWd^2DE^, LVIDs2DE, IVSd^MM^, LVFWd^MM^, LVIDs^MM^, AoD^MM^, and LAD^MM^. Finally, intra- and inter-operator repeatability was at least substantial for all measurements except LA-AP and LVFWd^2DE^.

## 4. Discussion

In the present study, we propose reference intervals and weight-based prediction intervals for a relatively large number of echocardiographic parameters, all evaluated in apparently healthy adult chickens of common companion flock breeds. This work expands on previously published echocardiographic standards for chickens [23,25,32], which focused primarily on left ventricular dimensions. The reference intervals for left ventricular dimensions proposed here are similar to (i.e., contain) those previously described. In addition, we report values for measures of left atrial size and left ventricular function, as well as Doppler-derived parameters. The methods used adhere to current guidelines for the determination of reference intervals, which dictate that the central 95% distribution of a measured variable be derived from a healthy reference sample that represents the animal population for which the reference interval will be used.

Given the differences in body size among various breeds of chickens, one goal of this study was to establish body weight-independent echocardiographic reference intervals. However, we found that of the many measurements with significant correlation to body weight, none were strongly correlated (i.e., no R^2^ values were ≥ 0.79; Table 2). Therefore, the reference intervals provided in Table 1 can be used for most companion chickens, whereas for birds with relative extremes of body weight (i.e., <1.5 kg or >2.5 kg), allometric data should be employed (Table 3).

Previously published echocardiographic measures of left ventricular chamber size in birds have been obtained in various ways, including from 2D long-axis images (as reported in psittacines [19]) or from 2D or MM recordings taken from a short-axis view (as previously used in chickens [23]). In the present study, we suggest reference intervals for measurements taken from long- or short-axis views, using 2D or MM echocardiography. Of note, variability (particularly between observers) tended to be greater for MM- versus 2D-derived measurements in the present study. Indeed, of the evaluated left ventricular measurements (2D- and MM-derived), repeatability and reliability were best, and reference and prediction intervals were most narrow, for LVL^2DE^, a measurement that has not been previously reported in chickens.

Valvular regurgitation was frequently identified in this cohort. Insufficiency of at least one valve was present in 23.8% of the chickens (30/126), and 5.5% (7/126) had insufficiency of two or more valves. The presence of trace to mild valvular regurgitation was not associated with a heart murmur or subjective chamber enlargement. Therefore, physiologic valvular insufficiency appears to be relatively common in adult chickens of common backyard breeds, similar to what is documented in numerous other species [50,51,52].

There was practical knowledge gained during planning and refinement of the echocardiographic protocol used in the present study. First, the featherless tract on either side of the keel was instrumental in avoiding ultrasound artifacts created by feather cuticles. In some female birds, egg follicles obscured normal imaging planes, necessitating adjustments to optimize views. Additionally, the pulmonary valve was challenging to visualize in the majority of chickens, limiting the interpretation of the outflow tract velocity and annulus measurements. Lastly, the three normal papillary muscles of the avian left ventricle—two septophilic and one septophobic [53]—can interfere with identification of the left ventricular free wall’s endocardial border, which likely impacted measurement variability and repeatability of this structure.

This study has limitations. The chickens used in this study are handled regularly and used for educational purposes; however, their diet, environment, handling practices, and stress exposure (e.g., stocking density) might not accurately represent conditions experienced by chickens in a typical home setting. While sexual dimorphism is a common feature of avian species and is associated with visible differences in size, feathering, and behavior [54], the effect of sex on echocardiographic variables was not investigated in this study due to uneven sex distribution and inadequate sample size. However, because sex and body weight are known co-variates, the use of the allometric data may aid in the accurate interpretation of larger male chicken studies.

All chickens were sedated using a standardized protocol to minimize their stress and facilitate image acquisition. Depending on the species and the agent(s) and route of their administration, certain echocardiographic measurements can be significantly impacted by sedation protocols [55,56,57]. To minimize the potential for these effects, the investigators chose drugs associated with few direct cardiovascular and respiratory effects based on mechanism of action and prior observations in chickens. All chickens were sedated so that any effects would be universally applied. Additionally, synchronized ECG was not utilized to minimize stimulation and associated stress, which may have precluded precise determination of cardiac cycle phases. Clinicians should note the potential influence of sedation and stress exposure on the reference intervals proposed and interpret results accordingly.

An additional limitation was uneven breed distribution due to reliance on a convenience sample, which might restrict the generalizability of findings to all non-production chickens. Of note, the second-most common breed included was the Athens Random Breed, accounting for one third of the final reference population. This is a mixed breed that was developed in 1956 from multiple strains—including Plymouth Rock, White Cornish, New Hampshire, Rhode Island Red, Barred Rock, White Leghorn, and Cornish, and therefore exhibits phenotypic characteristics similar to those commonly observed in hobby flocks [58].

## 5. Conclusions

This study proposes echocardiographic reference intervals for chickens of non-production breeds, which will support diagnostic accuracy and improve veterinary care and research by offering a robust tool for clinicians and researchers working with this population. In addition, body weight-based prediction intervals complement these general reference intervals, enabling more individualized assessment. Use of these values should also consider the sedation context and the specific characteristics of the study population to ensure appropriate clinical application.

## Figures and Tables

**Table 1:** Descriptive statistics and proposed reference intervals for echocardiographic reference intervals obtained from 126 healthy adult chickens. See text for abbreviations.

Echocardiographic Parameter	Sample Size	Median	95% CI of Median	Minimum-Maximum	95% RI: 2.5 Centile (90% CI) to 97.5 Centile (90% CI)
Right parasternal long-axis 5-chamber view					
LVLd^2DE^ (cm)	126	3.00	2.94 to 3.09	1.70 to 4.13	2.01 (1.70 to 2.31) to 3.91(3.85 to 4.13)
LVLs^2DE^ (cm)	126	1.99	1.87 to 2.08	0.90 to 3.27	1.10 (0.9 to 1.25) to 3.07 (2.93 to 3.27)
LVL-FS% (%)	126	33.65	32.35 to 35.84	12.80 to 52.80	14.55 (12.80 to 18.40) to 51.78 (49.10 to 52.80)
LA-AP (cm)	126	1.00	0.98 to 1.04	0.65 to 1.51	0.68 (0.65 to 0.72) to 1.41 (1.34 to 1.51)
LA-BA (cm)	126	1.345	1.30 to 1.40	0.78 to 2.01	0.84 (0.78 to 0.95) to 1.87 (1.78 to 2.01)
AoD^2DE,Lx^ (cm)	124	0.545	0.53–0.5646	0.33 to 0.82	0.39 (0.33 to 0.41) to 0.75 (0.69 to 0.82)
Right parasternal short-axis views					
IVSd^2DE^ (cm)	125	0.36	0.35 to 0.38	0.23 to 0.72	0.24 (0.23 to 0.27) to 0.60 (0.51 to 0.72)
LVIDd^2DE^ (cm)	125	1.51	1.47 to 1.57	0.84 to 2.29	1.04 (0.84 to 1.06) to 2.07 (1.87 to 2.29)
LVFWd^2DE^ (cm)	125	0.34	0.32 to 0.35	0.23 to 0.50	0.24 (0.23 to 0.26) to 0.48 (0.45 to 0.50)
LVIDs^2DE^ (cm)	125	1.09	1.05 to 1.12	0.36 to 1.83	0.64 (0.36 to 0.72) to 1.40 (1.33 to 1.83)
FS^2DE^ (%)	125	29.92	27.60 to 31.54	8.13 to 64.84	9.86 (8.13 to 10.86) to 44.72 (41.79 to 64.84)
LVAd (cm^2^)	125	1.33	1.26 to 1.42	0.58 to 2.27	0.62 (0.58 to 0.68) to 2.00 (1.10 to 2.27)
LVAs (cm^2^)	125	0.64	0.59 to 0.70	0.07 to 1.29	0.26 (0.07 to 030) to 1.24 (1.04 to 1.29)
LV FAC (%)	125	52.14	49.70 to 54.12	15.05 to 88.65	30.04 (15.05 to 34.04) to 68.90 (64.87 to 88.67)
IVSd^MM^ (cm)	120	0.40	0.37 to 0.41	0.22 to 0.70	0.26 (0.22 to 0.29) to 0.58 (0.50 to 0.70)
LVIDd^MM^ (cm)	120	1.52	1.43 to 1.56	0.72 to 2.21	0.87 (0.72 to 0.99) to 1.96 (1.87 to 2.21)
LVFWd^MM^ (cm)	120	0.41	0.40 to 0.43	0.24 to 0.68	0.27 (0.24 to 0.30) to 0.65 (0.58 to 0.68)
LVIDs ^MM^ (cm)	120	1.10	1.07 to 1.13	0.60 to 1.75	0.66 (0.60 to 0.74) to 1.57 (1.40 to 1.75)
FS^MM^ (%)	120	23.97	22.02 to 27.16	4.21 to 42.76	7.64 (4.21 to 10.68) to 41.20 (39.00 to 42.76)
EPSS (cm)	114	0.23	0.21 to 0.24	0.10 to 0.52	0.08 (0.06 to 0.11) to 0.38 (0.35 to 0.40)
AoD^2DE,Sx^ Swedish (cm)	125	0.78	0.76 to 0.80	0.53 to 1.13	0.55 (0.51 to 0.60) to 1.04 (0.97 to 1.13)
AoD^2DE,Sx^ Rishniw (cm)	125	0.79	0.77 to 0.83	0.50 to 1.07	0.54 (0.50 to 0.61) to 1.01 (0.99 to 1.07)
LA^2DE,Sx^ (cm)	125	1.28	1.25 to 1.31	0.65 to 1.67	0.74 (0.65 to 0.90) to 1.60 (1.53 to 1.67)
LA:Ao^2DE^ Swedish	125	1.61	1.57 to 1.65	1.03 to 2.08	1.16 (1.03 to 1.31) to 2.00 (1.92 to 2.08)
LA:Ao^2DE^ Rishniw	125	1.58	1.54 to 1.62	1.016 to 2.29	1.22 (1.02 to 1.27) to 2.08 (1.92 to 2.29)
AoD^MM^ (cm)	118	0.97	0.95 to 0.98	0.57 to 1.29	0.70 (0.67 to 0.74) to 1.23 (1.19 to 1.26)
LA^MM^ (cm)	118	1.54	1.51 to 1.60	0.85 to 1.89	1.10 (1.02 to 1.17) to 1.99 (1.92 to 2.05)
LA:Ao^MM^	118	1.58	1.54 to 1.60	1.05 to 2.13	1.11 (1.05 to 1.17) to 2.01 (1.95 to 2.08)
Left parasternal LV outflow view					
AV Vmax (m/s)	123	1.03	1.00 to 1.10	0.58 to 1.60	0.67 (0.58 to 0.71) to 1.52 (1.38 to 1.60)
AV VTI (cm)	123	7.83	7.57 to 8.23	4.33 to 11.97	4.90 (4.33 to 5.43) to 11.33 (10.83 to 11.97)
AV-AT (ms)	125	31.7	30.00 to 32.70	14.00 to 61.70	16.60 (14.00 to 18.30) to 54.245 (48.70 to 61.70)
AV-ET (ms)	126	102.15	100.25 to 105.00	69.7 to 144.80	79.42 (69.70 to 84.00) to 130.48 (124.30 to 144.80)
AV AT:ET	126	0.31	0.30 to 0.33	0.146 to 0.884	0.16 (0.15 to 0.19) to 0.55 (0.48 to 0.88)
LVOT Vmax (m/s)	124	1.08	1.03 to 1.13	0.633 to 1.86	0.74 (0.633 to 0.78) to 1.62 (1.53 to 1.86)
LVOT VTI (cm)	124	8.37	8.13 to 8.63	4.40 to 12.83	5.07 (4.40 to 5.83) to 11.92 (11.53 to 12.83)
Left parasternal 4-chamber view					
LVVd^SMOD^ (mL)	124	1.85	1.71 to 2.00	0.41 to 4.22	0.64 (0.41 to 0.77) to 4.07 (3.29 to 4.22)
LVVs^SMOD^ (mL)	124	0.59	0.52 to 0.69	0.09 to 2.03	0.15 (0.09 to 0.18) to 1.57 (1.22 to 2.03)
LV EF^SMOD^ (%)	124	68.15	65.91 to 69.28	45.87 to 85.76	48.47 (45.87 to 51.64) to 82.69 (81.90 to 87.31)
MV summated inflow Vmax (cm/s)	110	0.73	0.69 to 0.77	0.38 to 1.28	0.38 (0.33 to 0.43) to 1.08 (1.02 to 1.13)

**Table 2:** Results of the linear regression analyses describing how log_10_ of selected chamber measurements relate to log_10_ body weight in 126 healthy chickens.

Echocardiographic Chamber Measurement	N	Proportionality Constant (a)	SE of Y Estimate	Scaling Exponent (b)	SE of b	R^2^	*p* Value
Right parasternal 5-chamber view							
LVLd^2DE^ (cm)	126	2.447	0.008	0.324	0.0267	0.542	*p* < 0.0001
LVLs^2DE^ (cm)	126	1.443	0.015	0.495	0.048	0.457	*p* < 0.0001
LVL-FS% (%)	126	40.888	0.023	−0.382	0.075	0.173	*p* < 0.0001
LA-AP (cm)	126	0.853	0.013	0.266	0.044	0.229	*p* < 0.0001
LA-BA (cm)	126	1.126	0.013	0.265	0.043	0.235	*p* < 0.0001
AoD^2DE,Lx^ (cm)	124	0.431	0.008	0.382	0.026	0.639	*p* < 0.0001
Right parasternal short-axis views							
IVSd^2DE^ (cm)	125	0.307	0.015	0.279	0.049	0.207	*p* < 0.0001
LVIDd^2DE^ (cm)	125	1.323	0.013	0.186	0.044	0.126	*p* < 0.0001
LVFWd^2DE^ (cm)	125	0.296	0.011	0.212	0.039	0.192	*p* < 0.0001
LVIDs^2DE^ (cm)	125	0.852	0.014	0.321	0.046	0.284	*p* < 0.0001
FS^2DE^ (%)	125	35.003	0.027	−0.373	0.089	0.124	*p* = 0.0001
LVAd (cm^2^)	125	0.985	0.020	0.418	0.065	0.254	*p* < 0.0001
LVAs (cm^2^)	125	0.401	0.027	0.702	0.090	0.331	*p* < 0.0001
LV FAC (%)	125	58.614	0.017	−0.291	0.057	0.177	*p* < 0.0001
IVSd^MM^ (cm)	120	0.339	0.014	0.225	0.047	0.165	*p* < 0.0001
LVIDd^MM^ (cm)	120	1.176	0.014	0.248	0.047	0.190	*p* < 0.0001
LVFWd^MM^ (cm)	120	0.378	0.016	0.158	0.053	0.071	*p* = 0.0033
LVIDs ^MM^ (cm)	120	0.888	0.013	0.330	0.043	0.328	*p* < 0.0001
EPSS (cm)	114	0.201	0.025	0.211	0.082	0.056	*p* = 0.0111
AoD^2DE,Sx^ Swedish (cm)	125	0.627	0.007	0.344	0.023	0.653	*p* < 0.0001
AoD^2DE,Sx^ Rishniw (cm)	125	0.627	0.007	0.369	0.023	0.673	*p* < 0.0001
LA^2DE,Sx^ (cm)	125	1.019	0.011	0.314	0.035	0.389	*p* < 0.0001
AoD^MM^ (cm)	118	0.779	0.008	0.317	0.026	0.566	*p* < 0.0001
LA^MM^ (cm)	118	1.296	0.012	0.225	0.038	0.232	*p* < 0.0001
Left parasternal 4-chamber view							
LVVd^SMOD^ (mL)	124	1.109	0.029	0.748	0.095	0.337	*p* < 0.0001
LVVs^SMOD^ (mL)	124	0.890	0.041	0.929	0.136	0.287	*p* < 0.0001
LV EF^SMOD^ (%)	124	70.567	0.011	−0.092	0.036	0.051	*p* = 0.0115

**Table 3:** Body weight-based mean and 95% prediction intervals for echocardiographic variables in adult chickens derived from the allometric scaling parameters presented in Table 2.

Weight (kg)	0.5		1.0		1.5		2.0		2.5		3.0		3.5		4.0		4.5		5.0	
	Mean	95% PI	Mean	95% PI	Mean	95% PI	Mean	95% PI	Mean	95% PI	Mean	95% PI	Mean	95% PI	Mean	95% PI	Mean	95% PI	Mean	95% PI
LVLd^2DE^ (cm)	1.96	1.60 to 2.39	2.45	2.00 to 2.99	2.80	2.28 to 3.41	3.07	2.50 to 3.75	3.30	2.69 to 4.03	3.50	2.86 to 4.27	3.68	3.00 to 4.49	3.84	3.13 to 4.69	3.99	3.26 to 4.87	4.13	3.37 to 5.04
LVLs^2DE^ (cm)	1.03	0.71 to 1.48	1.45	1.00 to 2.08	1.78	1.22 to 2.54	2.05	1.41 to 2.93	2.29	1.58 to 3.27	2.51	1.73 to 3.58	2.70	1.86 to 3.87	2.89	1.99 to 4.13	3.06	2.11 to 4.38	3.23	2.22 to 4.61
LVL-FS% (%)	54.29	30.24 to 93.92	41.66	23.20 to 72.06	35.68	19.87 to 61.71	31.96	17.80 to 55.29	29.35	16.35 to 50.77	27.37	15.25 to 47.35	25.81	14.37 to 44.64	24.52	13.66 to 42.42	23.44	13.06 to 40.55	22.52	12.54 to 38.95
LA-AP (cm)	0.71	0.51 to 0.99	0.86	0.61 to 1.19	0.96	0.68 to 1.32	1.03	0.74 to 1.43	1.09	0.78 to 1.51	1.15	0.82 to 1.59	1.20	0.86 to 1.66	1.24	0.89 to 1.72	1.28	0.91 to 1.77	1.32	0.94 to 1.82
LA-BA (cm)	0.94	0.68 to 1.30	1.13	0.81 to 1.56	1.26	0.91 to 1.73	1.36	0.98 to 1.87	1.44	1.04 to 1.99	1.52	1.09 to 2.08	1.58	1.13 to 2.17	1.64	1.18 to 2.25	1.69	1.21 to 2.32	1.74	1.25 to 2.39
AoD^2DE,Lx^ (cm)	0.33	0.27 to 0.40	0.43	0.35 to 0.52	0.50	0.41 to 0.61	0.56	0.46 to 0.68	0.61	0.50 to 0.74	0.66	0.54 to 0.80	0.70	0.57 to 0.84	0.73	0.60 to 0.89	0.77	0.63 to 0.93	0.80	0.65 to 0.97
IVSd^2DE^ (cm)	0.26	0.17 to 0.37	0.31	0.21 to 0.45	0.35	0.24 to 0.50	0.38	0.26 to 0.54	0.40	0.27 to 0.57	0.42	0.29 to 0.60	0.44	0.30 to 0.63	0.46	0.31 to 0.66	0.47	0.32 to 0.68	0.49	0.33 to 0.70
LVIDd^2DE^ (cm)	1.17	0.83 to 1.62	1.33	0.95 to 1.84	1.44	1.02 to 1.99	1.51	1.08 to 2.10	1.58	1.13 to 2.19	1.63	1.16 to 2.26	1.68	1.20 to 2.33	1.72	1.23 to 2.39	1.76	1.26 to 2.44	1.80	1.28 to 2.49
LVFWd^2DE^ (cm)	0.26	0.19 to 0.34	0.30	0.22 to 0.40	0.32	0.24 to 0.43	0.34	0.25 to 0.46	0.36	0.27 to 0.48	0.38	0.28 to 0.50	0.39	0.29 to 0.52	0.40	0.30 to 0.53	0.41	0.30 to 0.55	0.42	0.31 to 0.56
LVIDs^2DE^ (cm)	0.69	0.48 to 0.96	0.86	0.60 to 1.20	0.98	0.69 to 1.37	1.07	0.75 to 1.51	1.15	0.81 to 1.62	1.22	0.86 to 1.71	1.28	0.90 to 1.80	1.34	0.94 to 1.88	1.39	0.98 to 1.95	1.44	1.01 to 2.02
FS^2DE^ (%)	46.52	23.13 to 88.78	35.93	17.87 to 68.57	30.89	15.36 to 58.95	27.75	13.80 to 52.96	25.54	12.70 to 48.73	23.86	11.86 to 45.53	22.53	11.20 to 42.99	21.43	10.66 to 40.90	20.51	10.20 39.15	19.72	9.81 to 37.64
LVAd (cm^2^)	0.75	. 045 to 1.20	1.00	0.61 to 1.60	1.18	0.72 to 1.90	1.33	0.81 to 2.14	1.47	0.89 to 2.35	1.58	0.96 to 2.54	1.69	1.02 to 2.71	1.78	1.08 to 2.86	1.87	1.14 to 3.01	1.96	1.19 to 3.14
LVAs (cm^2^)	0.25	0.13 to 0.49	0.41	0.20 to 0.79	0.55	0.27 to 1.05	0.67	0.33 to 1.28	0.78	0.39 to 1.50	0.89	0.44 to 1.71	0.99	0.49 to 1.90	1.09	0.54 to 2.09	1.18	0.58 to 2.27	1.27	0.63 to 2.44
LV FAC (%)	72.47	46.80 to 109.87	59.24	38.26 to 89.80	52.65	34.00 to 79.81	48.42	31.27 to 73.41	45.38	29.30 to 68.79	43.03	27.79 to 65.24	41.15	26.57 to 62.38	39.58	25.56 to 60.00	38.25	24.70 to 57.98	37.09	23.95 to 56.23
IVSd^MM^ (cm)	0.29	0.21 to 0.41	0.34	0.24 to 0.48	0.37	0.26 to 0.53	0.40	0.28 to 0.56	0.42	0.29 to 0.59	0.44	0.31 to 0.61	0.45	0.32 to 0.64	0.47	0.33 to 0.65	0.48	0.34 to 0.67	0.49	0.34 to 0.69
LVIDd^MM^ (cm)	1.00	0.70 to 1.40	1.18	0.83 to 1.67	1.31	0.92 to 1.84	1.41	0.98 to 1.98	1.49	1.04 to 2.09	1.55	1.09 to 2.19	1.62	1.13 to 2.27	1.67	1.17 to 2.35	1.72	1.20 to 2.42	1.76	1.24 to 2.48
LVFWd^MM^ (cm)	0.34	0.23 to 0.50	0.38	0.26 to 0.56	0.41	0.27 to 0.60	0.43	0.29 to 0.62	0.44	0.30 to 0.65	0.45	0.30 to 0.67	0.47	0.31 to 0.68	0.48	0.32 to 0.70	0.48	0.32 to 0.71	0.49	0.33 to 0.72
LVIDs ^MM^ (cm)	0.71	0.51 to 0.97	0.89	0.64 to 1.23	1.02	0.73 to 1.40	1.12	0.81 to 1.54	1.21	0.87 to 1.66	1.28	0.92 to 1.76	1.35	0.97 to 1.85	1.41	1.02 to 1.94	1.47	1.06 to 2.01	1.52	1.09 to 2.08
FS MM (%)	32.67	14.17 to 69.99	27.74	12.03 59.43	25.22	10.94 to 54.02	23.56	10.22 to 50.47	22.35	9.69 to 47.89	21.41	9.29 to 45.87	20.65	8.96 to 44.23	20.01	8.68 to 42.86	19.46	8.44 to 41.69	18.98	8.23 to 40.67
EPSS (cm)	0.18	0.10 to 0.31	0.21	0.11 to 0.36	0.22	0.12 to 0.39	0.24	0.13 to 0.41	0.25	0.14 to 0.43	0.26	0.14 to 0.45	0.27	0.15 to 0.47	0.27	0.15 to 0.48	0.28	0.16 to 0.49	0.29	0.16 to 0.50
AoD^2DE,Sx^ Swedish (cm)	0.50	0.42 to 0.59	0.63	0.53 to 0.74	0.72	0.61 to 0.86	0.80	0.67 to 0.94	0.86	0.73 to 1.02	0.92	0.77 to 1.09	0.97	0.81 to 1.14	1.01	0.85 to 1.20	1.05	0.89 to 1.25	1.09	0.92 to 1.29
AoD^2DE,Sx^ Rishniw (cm)	0.49	0.41 to 0.58	0.63	0.53 to 0.75	0.73	0.61 to 0.87	0.81	0.68 to 0.97	0.88	0.74 to 1.05	0.94	0.79 to 1.12	1.00	0.84 to 1.19	1.05	0.88 to 1.25	1.10	0.92 to 1.30	1.14	0.95 to 1.35
LA^2DE,Sx^ (cm)	0.82	0.63 to 1.07	1.02	0.78 to 1.33	1.16	0.89 to 1.51	1.27	0.97 to 1.65	1.36	1.04 to 1.77	1.44	1.10 to 1.88	1.52	1.16 to 1.97	1.58	1.21 to 2.06	1.64	1.25 to 2.13	1.69	1.29 to 2.20
AoD^MM^ (cm)	0.63	0.52 to 0.76	0.78	0.64 to 0.94	0.89	0.73 to 1.07	0.97	0.80 to 1.17	1.04	0.86 to 1.26	1.11	0.91 to 1.34	1.16	0.96 to 1.40	1.21	1.00 to 1.46	1.26	1.04 to 1.52	1.30	1.07 to 1.57
LA^MM^ (cm)	1.11	0.84 to 1.47	1.30	0.98 to 1.72	1.43	1.07 to 1.88	1.52	1.14 to 2.01	1.60	1.20 to 2.11	1.67	1.25 to 2.20	1.73	1.30 to 2.28	1.78	1.34 to 2.35	1.83	1.37 to 2.41	1.87	1.41 to 2.47
LVVd^SMOD^ (mL)	0.68	0.32 to 1.34	1.14	0.54 to 2.26	1.55	0.74 to 3.06	1.92	0.92 to 3.79	2.27	1.08 to 4.48	2.60	1.24 to 5.13	2.92	1.39 to 5.76	3.22	1.54 to 6.37	3.52	1.68 to 6.95	3.81	1.82 to 7.52
LVVs^SMOD^ (mL)	0.50	0.17 to 1.29	0.94	0.32 to 2.45	1.38	0.47 to 3.57	1.80	0.62 to 4.67	2.21	0.76 to 5.74	2.62	0.90 to 6.80	3.02	1.03 to 7.85	3.42	1.17 to 8.89	3.82	1.31 to 9.92	4.21	1.44 to 10.94
LV EF^SMOD^ (%)	75.53	57.53 to 98.32	70.86	53.98 to 92.25	68.27	52.01 to 88.87	66.49	50.65 to 86.55	65.14	49.62 to 84.79	64.06	48.80 to 83.39	63.15	48.11 to 82.21	62.38	47.52 to 81.21	61.71	47.01 to 80.33	61.12	46.56 to 79.56

**Table 4:** Intraobserver, interobserver, intraoperator, and interoperator agreement from 10 randomly selected chickens and associated echocardiographic studies.

Echo Measurement	Interobserver		Intraobserver		Intraoperator		Interoperator	
	ICC	95% CI	ICC	95% CI	ICC	95% CI	ICC	95% CI
LVLd^2DE^ (cm)	0.87	0.67, 0.96	0.85	0.64, 0.96	0.93	0.76, 0.98	0.70	0.38, 0.90
LVLs^2DE^ (cm)	0.83	0.58, 0.95	0.90	0.73, 0.97	0.96	0.85, 0.99	0.85	0.64, 0.96
LA-AP (cm)	0.63	0.26, 0.88	0.66	0.31, 0.89	0.54	−0.09, 0.86	0.65	0.31, 0.88
LA-BA (cm)	0.83	0.59, 0.95	0.84	0.61, 0.95	0.86	0.55, 0.96	0.73	0.38, 0.91
AoD^2DE,Lx^ (cm)	0.69	0.35, 0.90	0.90	0.69, 0.97	0.86	0.55, 0.96	0.92	0.79, 0.98
IVSd^2DE^ (cm)	0.76	0.46, 0.93	0.80	0.42, 0.95	0.82	0.44, 0.95	0.75	0.43, 0.92
LVIDd^2DE^ (cm)	0.71	0.38, 0.91	0.93	0.82, 0.98	0.65	0.10, 0.90	0.79	0.50, 0.94
LVFWd^2DE^ (cm)	0.36	−0.03, 0.75	0.79	0.53, 0.94	0.47	−0.08, 0.83	0.45	−0.5, 0.88
LVIDs^2DE^ (cm)	0.51	0.12, 0.82	0.96	0.88, 0.98	0.72	0.05, 0.94	0.85	0.61, 0.96
IVSd^MM^ (cm)	0.27	−0.17, 0.77	0.60	0.22, 0.87	0.86	0.44, 0.97	0.90	0.75, 0.98
LVIDd^MM^ (cm)	0.84	0.54, 0.97	0.94	0.85, 0.98	0.91	0.63, 0.98	0.78	0.49, 0.94
LVFWd^MM^ (cm)	0.26	−0.18, 0.77	0.30	−0.07, 0.71	0.70	0.13, 0.93	0.61	0.23, 0.88
LVIDs ^MM^ (cm)	0.60	0.13, 0.90	0.90	0.73, 0.97	0.91	0.65, 0.98	0.90	0.74, 0.97
AoD^2DE,Sx^ Swedish (cm)	0.64	0.28, 0.88	0.72	0.40, 0.91	0.77	0.01, 0.95	0.94	0.84, 0.99
AoD^2DE,Sx^ Rishniw (cm)	0.68	0.33, 0.90	0.78	0.49, 0.93	0.77	0.33, 0.94	0.95	0.86, 0.99
LA^2DE,Sx^ (cm)	0.80	0.52, 0.94	0.92	0.79, 0.98	0.95	0.81, 0.99	0.94	0.84, 0.99
AoD^MM^ (cm)	0.48	0.08, 0.81	0.53	0.38, 0.87	0.97	0.84, 0.99	0.89	0.67, 0.98
LA^MM^ (cm)	0.46	0.07, 0.80	0.58	0.21, 0.86	0.70	0.07, 0.93	0.77	0.43, 0.95
LVVd^SMOD^ (mL)	0.73	0.41, 0.91	0.80	0.55, 0.94	0.97	0.85, 0.99	0.95	0.79, 0.99
LVVs^SMOD^ (mL)	0.80	0.53, 0.94	0.85	0.56, 0.96	0.87	0.51, 0.97	0.97	0.87, 0.99

## Data Availability

The original contributions presented in this study are included in the article. Further inquiries can be directed to the corresponding author.

## Abbreviations

The following abbreviations are used in this manuscript:

ICC	Intraclass correlation coefficient
2D	Two-dimensional
MM	M-mode
CW	Continuous-wave
PW	Pulsed-wave
LV	Left ventricle
LA	Left atrium
Ao	Aorta
LVL	Left ventricular length
LA-AP	Left atrium, anterior to posterior
LA-BA	Left atrium, basiloapical
AoD	Aortic diameter
PVD	Pulmonary valve diameter
PV Vmax	Peak transpulmonary valve velocity
PV VTI	Transpulmonary velocity-time integral
PV-AT	Pulmonary valve acceleration time
PV-ET	Pulmonary valve ejection time
IVS	Interventricular septum
LVID	Left ventricular internal dimension
LVFW	Left ventricular free wall
FS	Fractional shortening
LVA	Left ventricular area
FAC	Fractional area change
EPSS	E-point-to-septal separation
LVV	Left ventricular volume
SMOD	Simpson’s method of disks
EF	Ejection fraction
AV Vmax	Peak transaortic valve velocity
AV-AT	Aortic valve acceleration time
AV-ET	Aortic valve ejection time
LVOT Vmax	Peak left ventricular outflow tract velocity

## Appendix A

**Table A1:** Descriptive statistics and proposed reference intervals for echocardiographic reference intervals for the pulmonary valve annulus and Doppler values obtained from healthy adult chickens. See text for abbreviations.

Echocardiographic Parameter	Sample Size	Median	95% CI of Median	Minimum-Maximum	95% RI: 2.5 Centile (90% CI) to 97.5 Centile (90% CI)
Right parasternal angled long axis					
PV annulusLx (cm)	25	0.53	0.49 to 0.58	0.35 to 0.75	0.30 (0.26 to 0.37) to 0.75 (0.68 to 0.81)
PV VmaxLx (m/s)	109	0.96	0.93 to 1.01	0.59 to 1.61	0.56 (0.51 to 0.62) to 1.34 (1.28 to 1.40)
PV VTILx (cm)	109	6.93	6.58 to 7.34	4.60 to 10.17	4.31 (4.00 to 4.16) to 9.46 (9.11 to 9.83)
PV ATLx (ms)	109	49.3	46.55 to 51.00	31.70 to 68.70	32.60 (30.67 to 34.75) to 64.00 (61.84 to 65.91)
PV ET Lx (ms)	109	103	99.17 to 108.30	67.00 to 141.70	71.23 (67.18 to 75.39) to 134.77 (131.59 to 138.86)
PV AT:ET Lx	109	0.48	0.46 to 0.49	0.31 to 0.68	0.33 (0.31 to 0.35) to 0.62 (0.60 to 0.64)
Right parasternal short axis					
PV annulus (cm)	32	0.49	0.43 to 0.52	0.32 to 0.69	0.29 (0.25 to 0.34) to 0.67 (0.62 to 0.72)
PV Vmax(m/s)	107	0.86	0.81 to 0.94	0.45 to 1.39	0.47 (0.42 to 0.52) to 1.26 (1.20 to 1.32)
PV VTI(cm)	108	6.47	6.13 to 6.63	3.37 to 9.60	3.90 (3.56 to 4.28) to 8.80 (8.43 to 9.15)
PV AT (ms)	108	48.50	46.70 to 51.00	32.3 to 112.00	30.24 (27.48 to 33.00) to 68.99 (63.26 to 74.94)
PV ET(ms)	108	102.15	97.90 to 105.13	70.3 to 162.00	66.49 (61.25 to 72.16) to 136.26 (130.26 to 142.45)
PV AT:ET	108	0.48	0.46 to 0.49	0.32 to 0.74	0.316 (0.30 to 0.34) to 0.64 (0.61 to 0.66)
